# A systematic review of clinical prediction rules to aid treatment selection in musculoskeletal physiotherapy practice

**DOI:** 10.1186/1753-6561-9-S1-A25

**Published:** 2015-01-14

**Authors:** P King, R Galvin, O’Sullivan, Z Bennett

**Affiliations:** 1 Royal College of Surgeons in Ireland, 123 St. Stephen’s Green, Dublin 2, Ireland; 2 HRB Centre for Primary Care Research, Department of General Practice, Royal College of Surgeons in Ireland, Dublin 2, Ireland

## Background

CPRs assist clinicians in making a diagnosis, prognosis or matching patients to optimal intervention based on a set of predictor variables that have been documented from a patient’s history, physical examination and in some situations available diagnostic tests. Within the field of musculoskeletal physiotherapy, a number of CPRs have been derived to target the most effective interventions for a given condition (Stanton 2010, [1]). The aim of this systematic review is to identify and critically appraise the CPRs in the area of musculoskeletal physiotherapy practice.

## Methods

A systematic literature search was conducted up to July 2013 and included PubMed, EBSCO and EMBASE. Citation tracking and hand searching of relevant journals were used as supplemental search strategies. Two review authors independently screened titles, keys words and abstracts of the references identified and excluded irrelevant studies. CPRs at any stage of their development (derivation, validation or impact analysis), consisting of >1 criterion, that were based on treatment selection for musculoskeletal conditions were included. CPRs were assessed for methodological quality using the McGinn criteria (2000) [2].

## Results

The literature search yielded 1347 articles after duplicates were removed. A total of 108 articles were retrieved and screened, of which 33 were included in the final review. Twenty studies were at the derivation stage of development. Eleven studies underwent narrow validation and only two studies had undergone impact analysis. In terms of the clinical domains, 14 CPRs focused on low back pain, seven focused on neck pain, 4 on patellofemoral pain, 4 on rheumatological conditions, two on ankle injuries, one on lateral epicondylitis and one on headache. The methodological quality of the studies varied, particularly with respect to study design and blinding of the assessors to the presence of the criteria contained in the CPRs.

## Conclusions

This review demonstrates that a number of CPRs have been derived for use in musculoskeletal practice, yet several of these have not been validated. Broad validation of these rules is required before consideration for use in clinical practice.

## References

[B1] Stanton TR Guyatt MJ Maher CG Koes BW Critical appraisial of clinical prediction rules that aim to optimize the treatment selection for musculoskeletal conditions Physical Therapy 2010 90 843 854 20413577 10.2522/ptj.20090233

[B2] McGinn TG Guyatt GH Wyer PC Naylor CD Steill IG Richardson WS for the Evidence Based Medicine Working Group User’s guide to the medical literature XXII: how to use articles about clinical decision rules Journal of the American Medical Assosciation 2000 284 79 84 10.1001/jama.284.1.79 10872017

